# Crystal structure of 2-[(di­chloro­methane)sulfon­yl]pyridine

**DOI:** 10.1107/S1600536814025148

**Published:** 2014-11-21

**Authors:** Zhiqiu Chen, Hembat Bolat, Xing Wan, Ya Li

**Affiliations:** a College of Chemistry and Chemical Engineering, Shanghai University of Engineering Science, 333 Longteng Road, Shanghai, People’s Republic of China

**Keywords:** crystal structure, sulfone, pyridine derivative, hydrogen bonding, π–π stacking

## Abstract

The asymmetric unit of the title compound, C_6_H_5_Cl_2_NO_2_S, contains two mol­ecules with similar conformations (r.m.s. overlay fit for the non-H atoms = 0.067 Å). Atoms attached to the pendent C*sp* ^3^—S bond are arranged in a staggered conformation with one of the Cl atoms *anti* to the C atom in the aromatic ring [C—S—C—Cl torsion angles = 178.41 (11) and −176.70 (13)°]. In the crystal, mol­ecules are linked by C—H⋯N and C—H⋯O hydrogen bonds, generating a three-dimensional network, and weak aromatic π–π stacking is also observed [centroid–centroid separation = 3.8902 (17) Å].

## Related literature   

For the biological activity of sulfone derivatives, see: Chen *et al.* (2012 ▶); Drews (2000 ▶); Raja *et al.* (2009 ▶). For the uses of halomethyl sulfone derivatives in organic synthesis, see: Li & Hu (2005 ▶); Prakash *et al.* (2013 ▶); Zhao *et al.* (2010 ▶). For the synthesis of the starting material, see: Kamiyama *et al.* (1988 ▶).
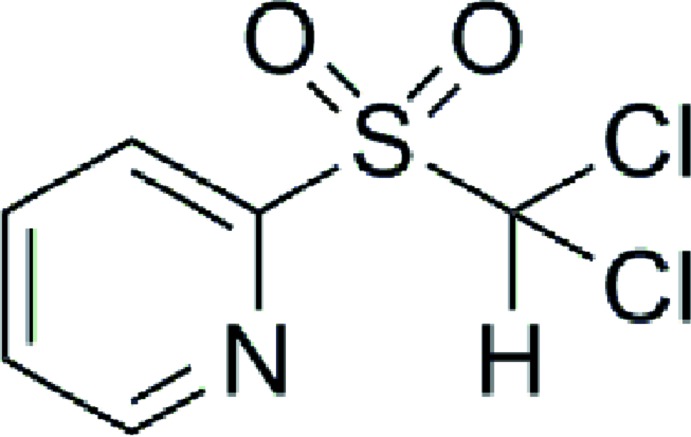


## Experimental   

### Crystal data  

C_6_H_5_Cl_2_NO_2_S
*M* *_r_* = 226.07
Monoclinic, 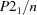

*a* = 9.9647 (10) Å
*b* = 12.2131 (11) Å
*c* = 15.7158 (15) Å
β = 108.483 (1)°
*V* = 1814.0 (3) Å^3^
*Z* = 8
Mo *K*α radiation
μ = 0.90 mm^−1^
*T* = 293 K
0.21 × 0.16 × 0.12 mm

### Data collection  

Bruker SMART CCD diffractometer
Absorption correction: multi-scan (*SADABS*; Bruker, 2007 ▶) *T*_min_ = 0.615, *T*_max_ = 0.746
10817 measured reflections
3570 independent reflections
2907 reflections with *I* > 2σ(*I*)
*R*_int_ = 0.034

### Refinement  

*R*[*F* ^2^ > 2σ(*F* ^2^)] = 0.037
*wR*(*F* ^2^) = 0.102
*S* = 1.03
3570 reflections
218 parameters
H-atom parameters constrained
Δρ_max_ = 0.37 e Å^−3^
Δρ_min_ = −0.37 e Å^−3^

### 

Data collection: *SMART* (Bruker, 2007 ▶); cell refinement: *SAINT* (Bruker, 2007 ▶); data reduction: *SAINT*; program(s) used to solve structure: *SHELXTL* (Sheldrick, 2008 ▶); program(s) used to refine structure: *SHELXL2013* (Sheldrick, 2008 ▶); molecular graphics: *SHELXTL*; software used to prepare material for publication: *SHELXTL*.

## Supplementary Material

Crystal structure: contains datablock(s) I. DOI: 10.1107/S1600536814025148/hb7316sup1.cif

Structure factors: contains datablock(s) I. DOI: 10.1107/S1600536814025148/hb7316Isup2.hkl

Supporting information file. DOI: 10.1107/S1600536814025148/hb7316Isup3.cml

**Fig. 1.**
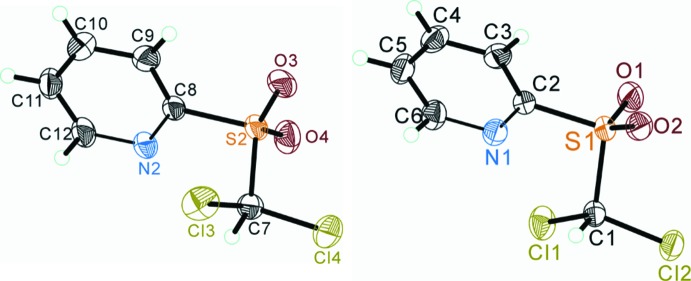
Molecular structure of the title compound. The displacement ellipsoids are drawn at the 50% probability level.

. DOI: 10.1107/S1600536814025148/hb7316fig1.tif
Mol­ecular structure of the title compound. The displacement ellipsoids are drawn at the 50% probability level.

CCDC reference: 1034519

Additional supporting information:  crystallographic information; 3D view; checkCIF report

## Figures and Tables

**Table 1:** Hydrogen-bond geometry (, )

*D*H*A*	*D*H	H*A*	*D* *A*	*D*H*A*
C1H1N2^i^	0.98	2.37	3.321(3)	163
C5H5O3^ii^	0.93	2.64	3.220(3)	121
C6H6O3^ii^	0.93	2.54	3.177(3)	126
C7H7N1^i^	0.98	2.36	3.303(3)	162
C11H11O1^iii^	0.93	2.64	3.292(3)	128

Symmetry codes: (i) 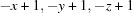; (ii) 
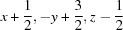
; (iii) 
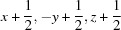
.

## supplementary crystallographic information

### S1. Comment

Sulfonyl groups are well-known for imparting biological activities to a lot of natural and unnatural molecules (Chen, *et al.*, 2012; Drews, 2000; Raja, *et al.*, 2009). Besides, halo­methyl sulfones have been widely used in the preparation of a wide variety of halogenated compounds (Li & Hu, 2005; Prakash, *et al.*, 2013; Zhao, *et al.*, 2010). Here, we report the crystal structure of 2-(di­chloro­methyl­sulfonyl)­pyridine, the title compound, which may find potential use in the synthesis of inter­esting chlorinated compounds.

### S2. Experimental

A mixture of sodium pyridine-2-sulfinate (660 mg, 4.0 mmol), chloro­form (10.0 ml) and 1N KOH (5.0 ml) was refluxed over 5h. Then the orgainc layer was separated and dried over anhydrous MgSO_4_. Evaporation of the solvent under vacuum, followed by flash chromatography, gave the title compound (white solid, 560 mg, 62 %). The obtained powder was recrystalized from ethyl acetate/hexane­(1:10) solution to give colourless prisms.

### S2.1. Refinement

The H atoms of the pyridine group were placed at calculated positions and treated as riding on the parent atoms, with Uiso(H) = 1.2Ueq(C). The di­chloro­methyl H atom was placed at calculated position with Uiso(H) = 1.2Ueq(C).

### Crystal data

**Table:**

C_6_H_5_Cl_2_NO_2_S	*F*(000) = 912
*M**_r_* = 226.07	*D*_x_ = 1.656 Mg m^−^^3^
Monoclinic, *P*2_1_/*n*	Mo *K*α radiation, λ = 0.71073 Å
*a* = 9.9647 (10) Å	Cell parameters from 4116 reflections
*b* = 12.2131 (11) Å	θ = 4.3–54.4°
*c* = 15.7158 (15) Å	µ = 0.90 mm^−^^1^
β = 108.483 (1)°	*T* = 293 K
*V* = 1814.0 (3) Å^3^	Prism, colorless
*Z* = 8	0.21 × 0.16 × 0.12 mm

### Data collection

**Table:**

Bruker SMART CCD diffractometer	2907 reflections with *I* > 2σ(*I*)
phi and ω scans	*R*_int_ = 0.034
Absorption correction: multi-scan (*SADABS*; Bruker, 2007)	θ_max_ = 26.0°, θ_min_ = 2.2°
*T*_min_ = 0.615, *T*_max_ = 0.746	*h* = −12→12
10817 measured reflections	*k* = −15→13
3570 independent reflections	*l* = −19→14

### Refinement

**Table:**

Refinement on *F*^2^	Hydrogen site location: inferred from neighbouring sites
Least-squares matrix: full	H-atom parameters constrained
*R*[*F*^2^ > 2σ(*F*^2^)] = 0.037	*w* = 1/[σ^2^(*F*_o_^2^) + (0.048*P*)^2^ + 0.7592*P*] where *P* = (*F*_o_^2^ + 2*F*_c_^2^)/3
*wR*(*F*^2^) = 0.102	(Δ/σ)_max_ = 0.001
*S* = 1.03	Δρ_max_ = 0.37 e Å^−^^3^
3570 reflections	Δρ_min_ = −0.37 e Å^−^^3^
218 parameters	Extinction correction: *SHELXL2013* (Sheldrick, 2013), Fc^*^=kFc[1+0.001xFc^2^λ^3^/sin(2θ)]^-1/4^
0 restraints	Extinction coefficient: 0.0286 (15)

### Special details

**Table:**

Geometry. All e.s.d.'s (except the e.s.d. in the dihedral angle between two l.s. planes) are estimated using the full covariance matrix. The cell e.s.d.'s are taken into account individually in the estimation of e.s.d.'s in distances, angles and torsion angles; correlations between e.s.d.'s in cell parameters are only used when they are defined by crystal symmetry. An approximate (isotropic) treatment of cell e.s.d.'s is used for estimating e.s.d.'s involving l.s. planes.

### Fractional atomic coordinates and isotropic or equivalent isotropic displacement parameters (Å^2^)

**Table:**

	*x*	*y*	*z*	*U*_iso_*/*U*_eq_	
S1	0.22031 (6)	0.33604 (5)	0.12351 (4)	0.04809 (18)	
S2	0.34445 (7)	0.72345 (5)	0.63426 (4)	0.05073 (19)	
Cl1	0.13628 (8)	0.50108 (5)	0.23115 (5)	0.0676 (2)	
Cl2	0.16196 (8)	0.27131 (5)	0.28379 (5)	0.0647 (2)	
Cl3	0.19229 (9)	0.60561 (8)	0.73732 (6)	0.0850 (3)	
Cl4	0.32582 (10)	0.81316 (7)	0.79958 (5)	0.0874 (3)	
N1	0.4188 (2)	0.47686 (16)	0.13024 (13)	0.0499 (5)	
N2	0.4604 (2)	0.53408 (16)	0.62759 (13)	0.0517 (5)	
O1	0.0757 (2)	0.32158 (16)	0.07175 (13)	0.0733 (6)	
O2	0.3169 (2)	0.24747 (14)	0.13497 (12)	0.0672 (5)	
O3	0.2167 (2)	0.77708 (15)	0.58491 (13)	0.0734 (6)	
O4	0.4775 (2)	0.77492 (15)	0.64783 (14)	0.0699 (5)	
C1	0.2303 (2)	0.37906 (17)	0.23599 (15)	0.0447 (5)	
H1	0.3296	0.3910	0.2713	0.054*	
C2	0.2870 (2)	0.45238 (18)	0.08275 (14)	0.0429 (5)	
C3	0.2047 (3)	0.5082 (2)	0.00872 (16)	0.0529 (6)	
H3	0.1126	0.4864	−0.0220	0.064*	
C4	0.2646 (3)	0.5977 (2)	−0.01787 (17)	0.0592 (6)	
H4	0.2135	0.6385	−0.0675	0.071*	
C5	0.4002 (3)	0.6258 (2)	0.02950 (19)	0.0607 (7)	
H5	0.4425	0.6861	0.0123	0.073*	
C6	0.4741 (3)	0.5646 (2)	0.10274 (18)	0.0578 (6)	
H6	0.5663	0.5851	0.1345	0.069*	
C7	0.3368 (3)	0.6905 (2)	0.74546 (16)	0.0523 (6)	
H7	0.4239	0.6524	0.7792	0.063*	
C8	0.3445 (2)	0.59091 (18)	0.58779 (15)	0.0454 (5)	
C9	0.2333 (3)	0.5566 (2)	0.51689 (17)	0.0596 (6)	
H9	0.1550	0.6010	0.4916	0.072*	
C10	0.2428 (3)	0.4520 (2)	0.48428 (19)	0.0691 (8)	
H10	0.1695	0.4238	0.4367	0.083*	
C11	0.3620 (3)	0.3912 (2)	0.52340 (19)	0.0648 (7)	
H11	0.3718	0.3217	0.5019	0.078*	
C12	0.4667 (3)	0.4344 (2)	0.59470 (19)	0.0612 (7)	
H12	0.5463	0.3918	0.6215	0.073*	

### Atomic displacement parameters (Å^2^)

**Table:**

	*U*^11^	*U*^22^	*U*^33^	*U*^12^	*U*^13^	*U*^23^
S1	0.0590 (4)	0.0370 (3)	0.0447 (3)	−0.0097 (2)	0.0115 (3)	−0.0018 (2)
S2	0.0659 (4)	0.0382 (3)	0.0499 (3)	0.0138 (3)	0.0209 (3)	0.0044 (2)
Cl1	0.0791 (5)	0.0434 (3)	0.0909 (5)	0.0082 (3)	0.0420 (4)	0.0000 (3)
Cl2	0.0849 (5)	0.0483 (4)	0.0719 (4)	−0.0087 (3)	0.0407 (4)	0.0051 (3)
Cl3	0.0800 (5)	0.1064 (7)	0.0806 (5)	−0.0173 (5)	0.0426 (4)	−0.0035 (5)
Cl4	0.1221 (7)	0.0714 (5)	0.0727 (5)	0.0256 (5)	0.0367 (5)	−0.0173 (4)
N1	0.0482 (11)	0.0488 (11)	0.0503 (11)	−0.0034 (9)	0.0123 (9)	0.0050 (9)
N2	0.0576 (12)	0.0422 (10)	0.0543 (11)	0.0135 (9)	0.0164 (9)	0.0040 (9)
O1	0.0736 (13)	0.0696 (12)	0.0613 (11)	−0.0329 (10)	−0.0005 (10)	0.0030 (9)
O2	0.1029 (15)	0.0415 (9)	0.0640 (11)	0.0094 (9)	0.0362 (11)	0.0002 (8)
O3	0.0935 (14)	0.0607 (11)	0.0617 (11)	0.0395 (10)	0.0183 (10)	0.0112 (9)
O4	0.0863 (14)	0.0513 (10)	0.0810 (13)	−0.0130 (9)	0.0390 (11)	−0.0032 (9)
C1	0.0474 (12)	0.0372 (11)	0.0512 (13)	−0.0037 (9)	0.0182 (10)	−0.0011 (9)
C2	0.0505 (13)	0.0383 (11)	0.0400 (11)	−0.0032 (9)	0.0145 (10)	−0.0009 (9)
C3	0.0541 (14)	0.0567 (14)	0.0444 (12)	0.0023 (11)	0.0104 (11)	0.0039 (11)
C4	0.0749 (18)	0.0531 (14)	0.0522 (14)	0.0128 (13)	0.0240 (13)	0.0148 (11)
C5	0.0749 (18)	0.0466 (13)	0.0696 (17)	−0.0037 (12)	0.0357 (15)	0.0081 (12)
C6	0.0539 (14)	0.0545 (14)	0.0655 (16)	−0.0086 (12)	0.0198 (12)	0.0043 (12)
C7	0.0574 (14)	0.0522 (13)	0.0493 (13)	0.0141 (11)	0.0195 (11)	0.0013 (11)
C8	0.0556 (13)	0.0390 (11)	0.0437 (12)	0.0071 (10)	0.0187 (10)	0.0023 (9)
C9	0.0640 (16)	0.0594 (15)	0.0512 (14)	0.0108 (12)	0.0124 (12)	0.0016 (12)
C10	0.083 (2)	0.0644 (17)	0.0554 (15)	−0.0077 (15)	0.0154 (14)	−0.0098 (13)
C11	0.093 (2)	0.0406 (13)	0.0681 (17)	0.0037 (13)	0.0359 (16)	−0.0039 (12)
C12	0.0751 (18)	0.0419 (13)	0.0687 (17)	0.0158 (12)	0.0259 (14)	0.0032 (12)

### Geometric parameters (Å, º)

**Table:**

S1—O2	1.4212 (19)	C2—C3	1.373 (3)
S1—O1	1.4235 (19)	C3—C4	1.372 (4)
S1—C2	1.772 (2)	C3—H3	0.9300
S1—C1	1.816 (2)	C4—C5	1.364 (4)
S2—O4	1.421 (2)	C4—H4	0.9300
S2—O3	1.4231 (19)	C5—C6	1.374 (4)
S2—C8	1.776 (2)	C5—H5	0.9300
S2—C7	1.818 (2)	C6—H6	0.9300
Cl1—C1	1.749 (2)	C7—H7	0.9800
Cl2—C1	1.756 (2)	C8—C9	1.364 (3)
Cl3—C7	1.746 (3)	C9—C10	1.390 (4)
Cl4—C7	1.743 (2)	C9—H9	0.9300
N1—C2	1.323 (3)	C10—C11	1.370 (4)
N1—C6	1.338 (3)	C10—H10	0.9300
N2—C8	1.323 (3)	C11—C12	1.371 (4)
N2—C12	1.332 (3)	C11—H11	0.9300
C1—H1	0.9800	C12—H12	0.9300
			
O2—S1—O1	120.03 (13)	C3—C4—H4	120.5
O2—S1—C2	109.84 (11)	C4—C5—C6	119.7 (2)
O1—S1—C2	108.69 (11)	C4—C5—H5	120.2
O2—S1—C1	105.72 (10)	C6—C5—H5	120.2
O1—S1—C1	108.98 (12)	N1—C6—C5	122.7 (2)
C2—S1—C1	102.05 (10)	N1—C6—H6	118.6
O4—S2—O3	120.59 (13)	C5—C6—H6	118.6
O4—S2—C8	110.07 (11)	Cl4—C7—Cl3	111.61 (13)
O3—S2—C8	108.19 (12)	Cl4—C7—S2	107.87 (13)
O4—S2—C7	105.97 (12)	Cl3—C7—S2	110.24 (13)
O3—S2—C7	108.84 (12)	Cl4—C7—H7	109.0
C8—S2—C7	101.49 (11)	Cl3—C7—H7	109.0
C2—N1—C6	115.8 (2)	S2—C7—H7	109.0
C8—N2—C12	116.0 (2)	N2—C8—C9	125.9 (2)
Cl1—C1—Cl2	112.46 (13)	N2—C8—S2	113.44 (17)
Cl1—C1—S1	109.92 (12)	C9—C8—S2	120.67 (18)
Cl2—C1—S1	106.89 (11)	C8—C9—C10	116.8 (2)
Cl1—C1—H1	109.2	C8—C9—H9	121.6
Cl2—C1—H1	109.2	C10—C9—H9	121.6
S1—C1—H1	109.2	C11—C10—C9	118.9 (3)
N1—C2—C3	125.8 (2)	C11—C10—H10	120.6
N1—C2—S1	113.34 (16)	C9—C10—H10	120.6
C3—C2—S1	120.84 (18)	C10—C11—C12	119.0 (2)
C4—C3—C2	116.9 (2)	C10—C11—H11	120.5
C4—C3—H3	121.5	C12—C11—H11	120.5
C2—C3—H3	121.5	N2—C12—C11	123.4 (2)
C5—C4—C3	119.1 (2)	N2—C12—H12	118.3
C5—C4—H4	120.5	C11—C12—H12	118.3
			
O2—S1—C1—Cl1	170.96 (12)	O4—S2—C7—Cl4	68.31 (15)
O1—S1—C1—Cl1	−58.75 (15)	O3—S2—C7—Cl4	−62.76 (16)
C2—S1—C1—Cl1	56.09 (14)	C8—S2—C7—Cl4	−176.70 (13)
O2—S1—C1—Cl2	−66.71 (14)	O4—S2—C7—Cl3	−169.59 (13)
O1—S1—C1—Cl2	63.58 (14)	O3—S2—C7—Cl3	59.34 (16)
C2—S1—C1—Cl2	178.41 (11)	C8—S2—C7—Cl3	−54.61 (15)
C6—N1—C2—C3	0.5 (4)	C12—N2—C8—C9	−0.3 (4)
C6—N1—C2—S1	−179.51 (18)	C12—N2—C8—S2	−179.28 (19)
O2—S1—C2—N1	−50.8 (2)	O4—S2—C8—N2	45.1 (2)
O1—S1—C2—N1	176.04 (17)	O3—S2—C8—N2	178.72 (18)
C1—S1—C2—N1	61.00 (19)	C7—S2—C8—N2	−66.86 (19)
O2—S1—C2—C3	129.1 (2)	O4—S2—C8—C9	−134.0 (2)
O1—S1—C2—C3	−4.0 (2)	O3—S2—C8—C9	−0.3 (2)
C1—S1—C2—C3	−119.0 (2)	C7—S2—C8—C9	114.1 (2)
N1—C2—C3—C4	−0.4 (4)	N2—C8—C9—C10	0.6 (4)
S1—C2—C3—C4	179.66 (18)	S2—C8—C9—C10	179.4 (2)
C2—C3—C4—C5	0.2 (4)	C8—C9—C10—C11	−1.1 (4)
C3—C4—C5—C6	−0.1 (4)	C9—C10—C11—C12	1.5 (4)
C2—N1—C6—C5	−0.5 (4)	C8—N2—C12—C11	0.7 (4)
C4—C5—C6—N1	0.3 (4)	C10—C11—C12—N2	−1.3 (4)

### Hydrogen-bond geometry (Å, º)

**Table:**

*D*—H···*A*	*D*—H	H···*A*	*D*···*A*	*D*—H···*A*
C1—H1···N2^i^	0.98	2.37	3.321 (3)	163
C5—H5···O3^ii^	0.93	2.64	3.220 (3)	121
C6—H6···O3^ii^	0.93	2.54	3.177 (3)	126
C7—H7···N1^i^	0.98	2.36	3.303 (3)	162
C11—H11···O1^iii^	0.93	2.64	3.292 (3)	128

Symmetry codes: (i) −*x*+1, −*y*+1, −*z*+1; (ii) *x*+1/2, −*y*+3/2, *z*−1/2; (iii) *x*+1/2, −*y*+1/2, *z*+1/2.
