# Supplementary figures and images for: Crystal structure of 2-[(di­chloro­methane)sulfon­yl]pyridine
Source: Acta Crystallogr Sect E Struct Rep Online. 2014 Nov 21;70(Pt 12):o1272. doi: 10.1107/S1600536814025148 (PMC4257385; doi:10.1107/S1600536814025148)

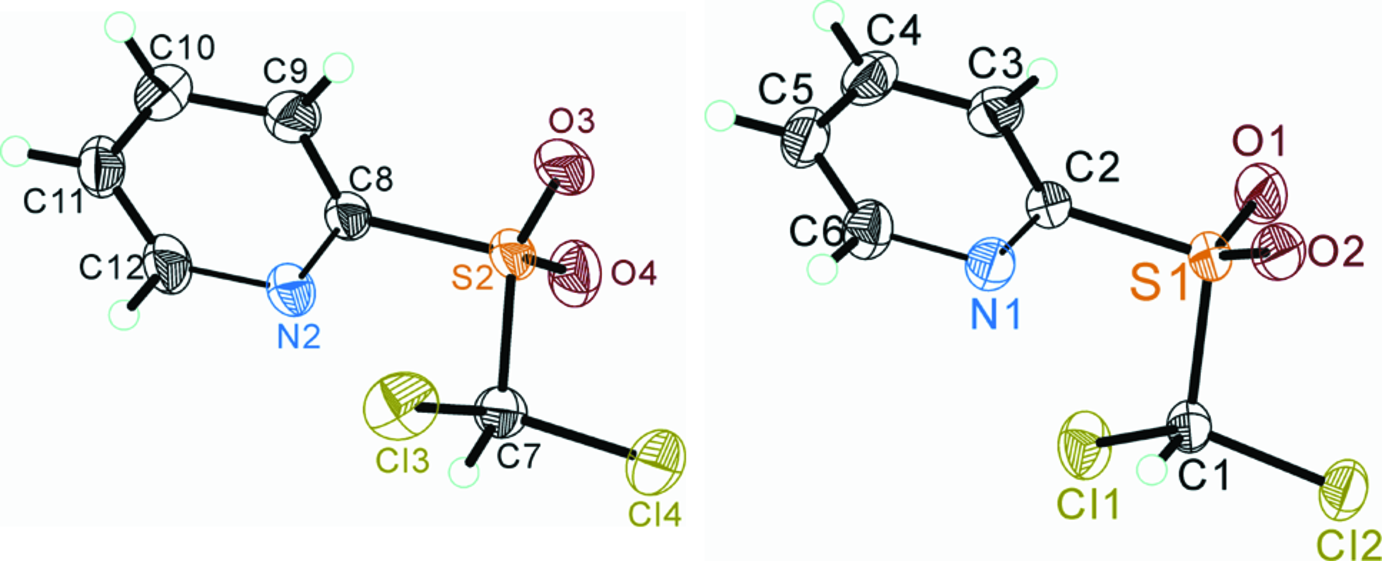

Supplement: Supplementary file 4 [file e-70-o1272-fig1.tif]
